# Surgical treatment of primary cardiac tumors in children

**DOI:** 10.1007/s11748-023-01958-z

**Published:** 2023-07-29

**Authors:** Jian Fu, HongBo Li, ZhengXia Pan, Chun Wu, YongGang Li, Gang Wang, JiangTao Dai, Lu Zhao

**Affiliations:** Department of Cardiothoracic Surgery, National Clinical Research Center for Child Health and Disorders, Ministry of Education Key Laboratory of Child Development and Disorders Chongqing Key Laboratory of Pediatrics, Children’s Hospital of Chongqing Medical University, No.136, Zhongshan 2nd Road, Yuzhong District, Chongqing, 400014 China

**Keywords:** Children, Primary, Cardiac tumor, Surgery, Effect

## Abstract

**Objective:**

Summarizing the treatment experience of primary cardiac tumors in children.

**Methods:**

The date of 24 children with primary cardiac tumors who underwent surgery in our department from July 2003 to September 2022 was collected and analyzed treatment efficacy.

**Results:**

All patients completed the surgery successfully, including 21 cases of complete tumor resection, 2 cases of partial tumor resection, and 1 case of tumor biopsy. The location: 5 cases in the right atrium, 5 cases in the right ventricle, 6 cases in the left atrium, 6 cases in the left ventricle, 1 case in the left, right ventricle and ventricular septum, and 1 case in the ventricular septum. 23 cases were benign: 11 cases of myxoma, 7 cases of fibroma, 3 cases of rhabdomyoma, 1 case of infantile capillary hemangioma, and 1 case of lipoma. There was 1 case of borderline or malignant tumor. 23 patients were discharged successfully, 1 patient died of cardiac failure on the first day after operation. Follow-up was done from 5 months to 19 years and 2 months, 2 cases were lost to follow-up, and 1 case died of cardiac failure in the second year after operation due to severe mitral regurgitation. There was 1 case of tumor biopsy with space-occupying lesion gradually shrinking during follow-up. The prognosis of another 19 children with complete or partial tumor resection was good. There was no recurrence, enlargement, or reoperation of the tumor during the follow-up period.

**Conclusions:**

Primary cardiac tumors in children are mostly benign. Surgery is effective, but the timing of surgery depends on the patient's condition.

## Introduction

Primary cardiac tumor (PCT) is a rare cardiac disease, particularly in infants and children and has an incidence of approximately 0.03%–0.1% [1]. In recent years, there has been an increase in research on cardiac tumors at home and abroad, but reports in the literature have focused mainly on adults. Reports of surgical treatment of primary cardiac tumors in children are rare and mostly case reports [2–4]. Here, we retrospectively review the clinical data and outcomes of children with primary cardiac tumors who underwent surgical treatment in our department over the past 19 years to summarize the experience of surgical diagnosis and treatment of pediatric primary cardiac tumors.

## Materials and methods

### Clinical information

Information on twenty-four children with PCT, including 12 males and 12 females aged 0.33–153 months (52.63 ± 53.99 months) and weighing 2.95–65 kg (19.46 ± 16.34 kg), who were surgically treated at the Department of Thoracic and Cardiac Surgery, Children's Hospital of Chongqing Medical University, from July 2003 to September 2022, was collected. The reasons for visiting were different and were as follows: productive examination or physical examination found a space-occupying lesion (7 cases), respiratory infection (6 cases), heart murmur (4 cases), cerebral infarction or limb embolism symptoms (3 cases), palpitations, chest tightness and chest pain (2 cases), or digestive disease (2 cases). All children completed echocardiography (ECHO), 11 underwent perfect cardiac magnetic resonance imaging (MRI), and 8 underwent perfect cardiac computed tomography (CT). The tumor size was approximately 0.2×0.3×0.2–7×8×5 cm, with the location being the right cardiac system in 10 patients, the left cardiac system in 12 patients, the interventricular septum in 1 patient, and multiple locations in 1 patient. The preoperative electrocardiogram (ECG) was abnormal in 15 patients, showing mainly sinus arrhythmia, right bundle branch block, ST-T segment changes, and T wave changes, among others (See Tables 1, 2).

**Table 1:** General data of 24 children with primary cardiac tumors

	Pathology	Age (month)	Gender	Weight (kg)	Preoperative examination	Intracardiac malformations	ECG
1	Myxoma	72	Female	19	ECHO	–	Sinus arrhythmia
2	Myxoma	83	Male	19.5	ECHO	PFO	Sinus arrhythmia
3	Myxoma	97	Male	21	ECHO + MRI	MR	Sinus tachycardia, ST-T segment changes
4	Myxoma	2	Male	3.6	ECHO	PFO + TR	Normal
5	Myxoma	5.5	Female	9	ECHO	–	Normal
6	Fibroma	66	Female	18	ECHO + CTA + MRI	RVOTO	Sinus arrhythmia, Incomplete right bundle branch block
7	Fibroma	34	Female	15	ECHO + CTA + MRI	RVOTO	Normal
8	Myxoma	150	Female	42	ECHO	–	Right axis deviation, Low voltage propensity
9	Myxoma	105	Female	20	ECHO	–	Sinus tachycardia, right atrial enlargement, T wave changes
10	Fibroma	5.5	Female	6.5	ECHO + MRI	–	Normal
11	Lipoma	18	Female	9	ECHO + MRI	LVOTO	Normal
12	Infantile capillary hemangioma	5	Female	8	ECHO + MRI	PFO	Normal
13	Rhabdomyoma	1	Male	5	ECHO + MRI	PFO + TR	Complete right bundle branch block with left axis deviation
14	Myxoma	29	Male	11	ECHO + MRI	PFO	Normal
15	Rhabdomyoma	9	Male	8.5	ECHO + MRI	RVOTO + PFO	Sinus arrhythmia, intermittent pre excitation syndrome
16	Myxoma	153	Female	45	ECHO + CTA	ASD + MR	Sinus bradycardia, ST-T segment changes
17	Rhabdomyoma	3	Male	7	ECHO	–	ST-T segment changes
18	Fibroma	4.5	Female	7	ECHO	TR	T wave changes, QT prolongation
19	Myxoma	76	Male	20	ECHO + MRI	–	Sinus arrhythmia
20	Fibroma	152	Male	33	ECHO + MRI	–	Sinus arrhythmia, T wave changes
21	Fibroma	128	Female	51	ECHO	–	Intraventricular block
22	Myxoma	53	Male	19	ECHO	Ebstein’s anomaly	Bi atrial anomalies
23	Fibroma	11	Male	8.5	ECHO + CTA	–	Third degree AV block
24	Reticular hemangioendothelioma/ Dabska tumor	0.33	Male	2.95	ECHO + CTA	ASD + PDA	Sinus rhythm, low voltage propensity

*MR* mitral regurgitation, *TR* tricuspid regurgitation, *PFO* patent foramen ovale, *ASD* atrial septal defect, *PDA* patent ductus arteriosus, *RVOT* right ventricular outflow tract, *LVOT* left ventricular outflow tract, *RVOT*O right ventricular outflow tract obstruction, *LVOTO* left ventricular outflow tract obstruction

**Table 2:** Surgical data of 24 children with primary cardiac tumors

	Pathology	Position	Size (cm)	Manifestation/surgical indication	Resection approaches	Modus operation
1	Myxoma	LV	1.8×1.5×1	Swing with cardiac cycle, shedding risk	AO	CPB + total resection (stalk)
2	Myxoma	LA	4×3.5×1	Limited mitral valve opening	AS	CPB + total resection (stalk)
3	Myxoma	LA	4×3.5×2	Severe regurgitation of mitral valve	AS	CPB + total resection + mitral valve repair (stalk)
4	Myxoma	RA	0.2×0.3×0.2	Swing with cardiac cycle, shedding risk, severe tricuspid regurgitation	RA	CPB + total resection + tricuspid valve repair(stalk)
5	Myxoma	RA	2×2×1.8	Swing with cardiac cycle, shedding risk, limited tricuspid valve opening	RA	CPB + total resection (stalk)
6	Fibroma	RV	6.3×5×4.5	RVOTO	RA + RVOT	CPB + total resection (enucleation)
7	Fibroma	IVS	8×5×4	RVOTO	AS + RV	CPB + partial resection (enucleation)
8	Myxoma	LA	6×5×4	Cerebral embolism	AS	CPB + total resection (stalk)
9	Myxoma	LA	6×7×5.5	visceral embolism、limited mitral valve opening	AS	CPB + partial resection (stalk)
10	Fibroma	LV	5×4×2	Reduced LV compression volume, heart failure	Light-sided transthoracic	biopsy
11	Lipoma	LV + RV + IVS	Multiple,Maximum Approx 1.2×0.6×1	LVOTO	AO	CPB + partial resection (enucleation)
12	Infantile capillary hemangioma	RA	1.5×2×1	Limited tricuspid valve opening	RA	CPB + total resection (some atrial appendage tissues were excised simultaneously)
13	Rhabdomyoma	RV	4×3×2.5	Severe tricuspid regurgitation	RVOT	CPB + total resection + tricuspid valve repair(enucleation)
14	Myxoma	LA	4.9×3.7×3.2	Cerebral embolism	AS	CPB + total resection (stalk)
15	Rhabdomyoma	RV	2.5×2.2×2	RVOTO	RA + RVOT	CPB + total resection (enucleation)
16	Myxoma	LA	6×5×1.5	Cerebral embolism, Severe regurgitation of mitral valve	AS	CPB + total resection + mitral valve repair(stalk)
17	Rhabdomyoma	LA	5×4×3	Reduced LA compression volume, heart failure	LA	CPB + total resection (enucleation)
18	Fibroma	RV	7×5×4	Severe tricuspid regurgitation	RV	CPB + total resection + tricuspid valve repair(enucleation)
19	Myxoma	LV	3×3×2.5	Cerebral embolism	LV	CPB + total resection (stalk)
20	Fibroma	LV	5.6×4.1×3.9	Reduced LV compression volume, heart failure	LV	CPB + total resection (enucleation)
21	Fibroma	LV	7×8×5	Reduced LV compression volume, heart failure	LV	CPB + total resection (enucleation)
22	Myxoma	RV	3×1×1	Severe tricuspid regurgitation	RA	CPB + total resection + tricuspid valve repair(stalk)
23	Fibroma	LV	6.6×5.6×4.5	Refractory arrhythmia	LV	CPB + total resection (enucleation)
24	Reticular hemangioendothelioma/ Dabska tumor	RA	1.8×1.5×1	Pericardial effusion, heart failure	RA	CPB + total resection (some right atrial appendage tissues were excised simultaneously)

*RA* right atrium, *LA* left atrium, *RV* right ventricle, *LV* left ventricle, *AS* atrial septum, *IVS* interventricular septum, *AO* aorta, *CPB* cardiopulmonary bypass

### Surgical indications

(1) Refractory arrhythmia, (2) Embolism or potential embolism risk, (3) Significant impairment of cardiac valve function, (4) ventricular outflow or inflow obstruction, (5) Heart failure or obstruction caused by decreased cardiac volume, (6) Rapid growth or suspected malignancy.

### Surgical approach

Twenty-three children in this series underwent surgical resection of tumor lesions with general anesthesia and hypothermic cardiopulmonary bypass. All were thoracotomized at the median incision, and CPB was established by routine cannulation of the aorta, superior vena cava (SVC), and inferior vena cava (IVC). The aortic root was perfused with cardioprotective solution. Different routes were chosen to remove the tumor. A single right atrial incision was used in 5 patients, a single left atrial incision was used in 1 patient, a left ventricular approach was used in 4 patients, a right ventricular approach was used in 1 patient, a right atrial and right ventricular outflow tract approach was used in 2 patients, a right ventricular outflow tract approach was used in 1 patient, and an aortic approach was used in 2 patients. The atrial septal incision approach was performed in 6 patients. The atrial septal and right ventricular incision approaches were performed in 1 patient. In 21 of these patients, the tumor was completely resected, and other 2 children’s tumors were partially resected. During the same period, six and two patients underwent PFO repair (*n* = 6) and MR-plasty (*n* = 2), respectively. (In case 3, the tumor was connected to the anterior mitral valve, and the anterior and posterior valvular bodies were thickened and coiled, underwent double-orifice operation. In case 16, the tumor was attached to the left atrial surface at the root of the anterior mitral valve and was interrupted with a cushion suture at the junction of the anterior and posterior valves.) Four patients underwent TR-plasty (*n* = 4). (In case 4, the tumor was attached to the right atrioventricular septal valve, which was sutured at the junction of septal and prevalvular valves. In case 13, the tumor involved the entire inferior papillary muscle, the tricuspid valve was doubly fenestrated by the interrupted suture between its free margin and the anterior margin of the septal valve after the chordae tendineae of the anterior valve was sheared off by the resected tumor during the operation. In case 18, the tricuspid valve was compressed and convex into the right atrium, and chordae tendineae and papillary muscles were partially damaged and sutured at the junction of the septal and anterior valves. In case 22, the tricuspid annulus enlarged, and there were scar-like changes at the lower border of the septal valve, thick inferior papillary muscles, and short chordae tendineae of second order. With a mattress varus suture at the posterior septal valve junction, the inferior border of the immobile septum was turned over to the right ventricular surface to recreate the posterior septal valve junction, the superior border of the annulus was retracted, the inferior papillary muscle was incised, and the anterior flap fissure was interrupted and sutured). Additionally, three patients underwent RVOTO dredge (*n* = 3), two underwent ASD repair (*n* = 2), one underwent LVOTO dredge (*n* = 1), one underwent Ebstein's anomaly correction (*n* = 1), and one underwent PDA ligation (*n* = 1). After removal of the tumor, patients with a large atrial/ventricular septal defect were repaired with a bovine pericardial patch or autogenous pericardium, and those with outflow tract obstruction were treated with an expanded bovine pericardial or autogenous pericardial patch. In the other child, because the tumor lesion was located in the lateral wall of the left ventricle, which was closely related to the coronary artery, the right lateral decubitus position was taken, and the tumor was biopsied through a transthoracic incision on the left side under cardiopulmonary bypass. The intraoperative resected lesion specimens were sent to the Department of Pathology for histological detection. This study was approved by the Ethics Committee of our hospital (approval number: ethics (research) No. 539 in 2022). This study involving human participants was conducted in accordance with the 1964 Helsinki Declaration and its later amendments and comparable ethical standards.

### Statistical methods

All data were processed and analyzed using SPSS 22 statistics, and count data are presented as the number of cases (percentage) and analyzed with the *χ*2 test. Measurement data are expressed as the mean ± standard deviation (mean ± SD) and were analyzed with the *t* test. *P* < 0.05 was considered statistically significant.

## Results

### Surgery results

The procedure was successfully completed in all 24 children, including 23 who underwent complete or partial resection of the tumor with the assistance of cardiopulmonary bypass, with an operative time of 140–339 min (196.13 ± 58.23 min), cardiopulmonary bypass time of 43–255 min (97.39 ± 50.37 min), and aortic occlusion time of 10–199 min (61.52 ± 46.13 min). One of these children, a newborn with a right atrial tumor, presented with massive pericardial effusion, respiratory failure, and multiple intracardiac malformations immediately before surgery and died on postoperative day 1 due to intractable low cardiac output, systemic multi-visceral dysfunction, and circulatory failure. One child had severe mitral valve regurgitation as case 3, which was treated by repeated plastic surgery after resection of the tumor, but the effect was poor. The patient ultimately underwent surgery for a double-orifice mitral valve. Postoperative review of ECHO still suggested that mitral regurgitation was severe, and mitral valve replacement was recommended, but the family members of the child refused. In one child, the tumor was closely related to the papillary muscles and chordae tendineae of the tricuspid valve as case 13, and the patient had moderate to severe tricuspid regurgitation after resection of the tumor, with significant improvement in tricuspid regurgitation after using the double-orifice technique. In one child, the tumor lesion was located in the lateral wall of the left ventricle as case 10, which is closely related to the muscles of the coronary artery and ventricular wall, and the lesion was large, making resection difficult; therefore, a tumor biopsy was taken.

### Tumor location and pathology

Among the 24 children with cardiac tumors, right atrial tumors (*n* = 5), right ventricular tumors (*n* = 5), left atrial tumors (*n* = 6), ventricular tumors (*n* = 6), multiple left and right ventricles and interventricular septum (*n* = 1), and ventricular septum (*n* = 1) were observed.

**Fig. 1:**
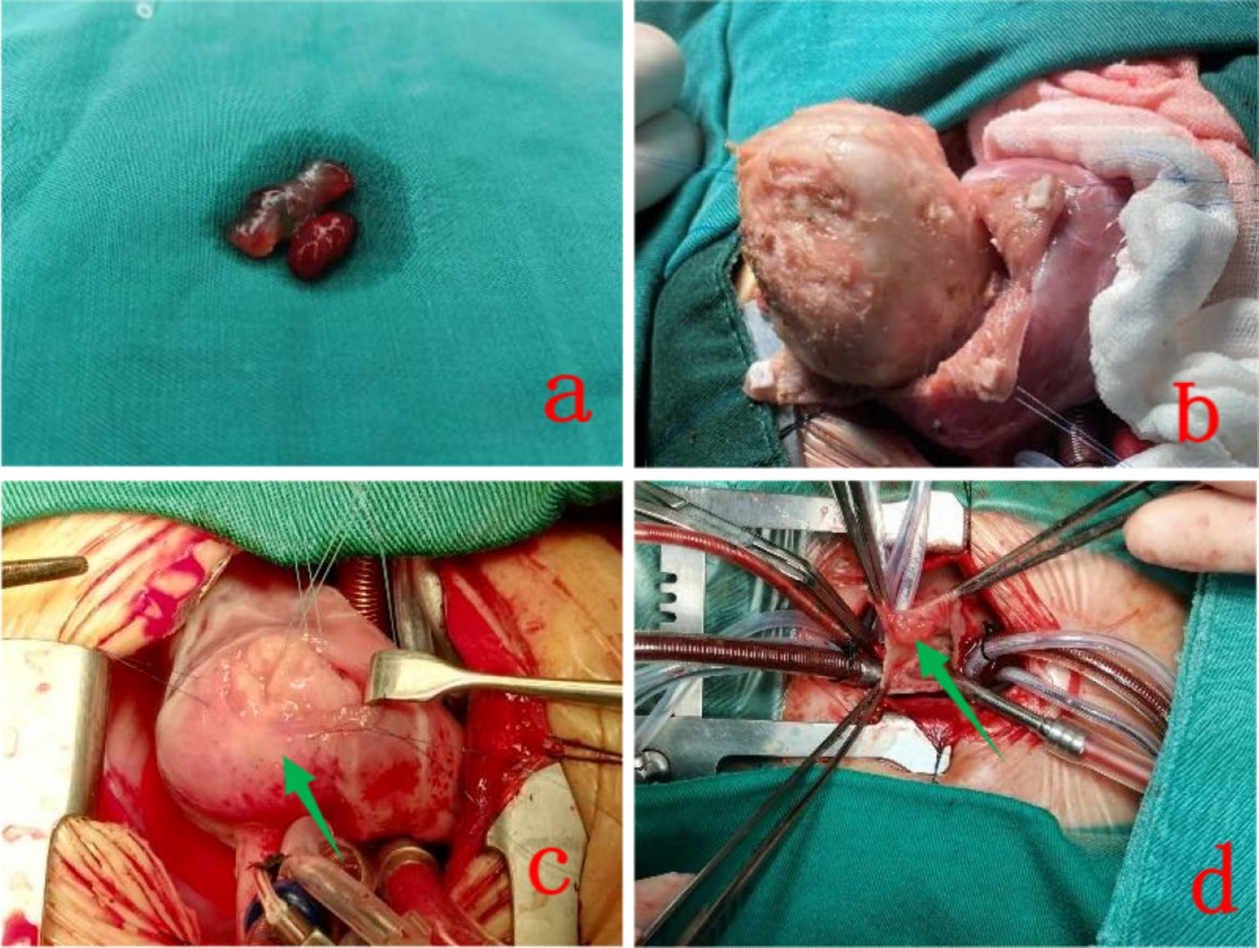
Intraoperative picture of cardiac oncology. Myxomas (**a**), fibromas (**b**), rhabdomyomas (**c**), Reticular hemangioendothelioma or Dabska tumor (**d**)

**Fig. 2:**
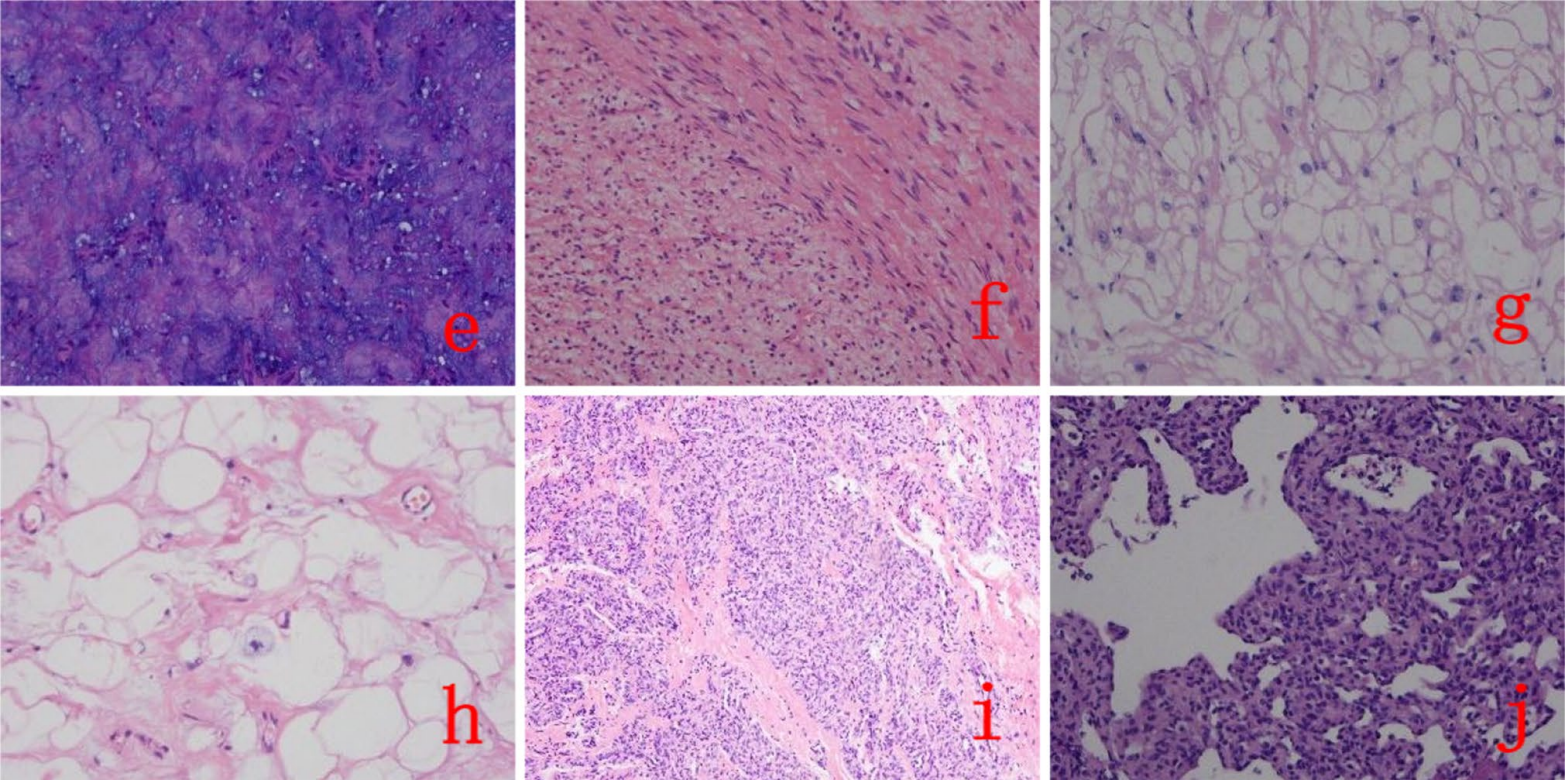
The cardiac tumor pathology picture. Myxomas (**e**) (HE, × 200), fibromas (**f**) (HE, × 200), rhabdomyomas (**g**) (HE, × 200), lipomas (**h**) (HE, × 200), infantile capillary hemangiom s (**i**) (HE, × 100), Reticular hemangioendothelioma/Dabska tumor (**j**) (HE, × 200)]

Postoperative pathology was benign in 23 cases: myxomas occurred in 11 patients (45.82%), fibromas in 7 patients (29.17%), rhabdomyomas in 3 patients (12.5%), infantile capillary hemangiomas in 1 patient (4.17%) and lipomas in 1 patient (4.17%). One patient had a borderline or low-grade tumor: reticular hemangioendothelioma or Dabska tumor 1 (4.17%) (See Figs. 1, 2).

### Postoperative complications and hospitalization

Complications were observed in 4 children, and the complication rate was approximately 16.67%. One child developed chylothorax postoperatively, improved and was discharged after treatment by fasting and nutritional support. One child developed capillary leak syndrome after surgery, and he was discharged with better outcomes with fluid replacement and colloid supplementation. A low cardiac output syndrome was observed in 2 children, 1 of whom recovered after improvement in cardiac function with intensive treatment and diuresis. Another patient died on the first postoperative day due to intractable low cardiac output that was difficult to correct, combined with systemic multi-visceral impairment. The remaining 20 children all recovered uneventfully after surgery and were discharged in better condition.

Except for the deceased child, the remaining 23 children spent 2–168 h (32.39 ± 52.15 h) with a postoperative ventilator, 2–9 days (3.83 ± 2.17 d) in intensive care, and 8–50 days (14.61 ± 8.28 d) in the hospital postoperatively.

### Follow-up

The follow-up was conducted from 5 months to 19 years and 2 months by telephone or outpatient visits, and the overall prognosis was good. Two children were lost to follow-up due to social factors, resulting in a follow-up rate of approximately 91.3% (21/23). One child had severe mitral valve regurgitation after surgery with suboptimal results of conservative treatment, and the guardian refused mitral valve replacement and died in the second postoperative year due to an acute attack of chronic heart failure. One child’s tumor was decreased during follow-up after biopsy. The remaining 19 children with complete and partial tumor resection had a favorable outcome, and there was no recurrence, enlargement, or reoperation during follow-up.

## Discussion

In recent years, neoplastic diseases have increased yearly, but the regeneration ability of myocardial tissue is poor relative to other tissues and organs of the human body, and few tumor lesions occur there. Cardiac tumors were first reported in 1934 and can be divided into benign tumors and malignant tumors according to the nature of the tumor, with the vast majority of them being benign lesions that account for more than 90% of cases [5]. Myxomas, fibromas, rhabdomyomas, and so on were included, with myxomas and rhabdomyomas being the most common [6–8]. Myxomas were the most common in this group of cases, followed by fibromas, and rhabdomyomas had the third highest incidence. There are some differences with related literature reports, and we consider it to be related to the small number of cases.

Cardiac tumors are mostly asymptomatic or nonspecific. They are often found incidentally during the examination of other diseases, and with the popularity of labor and improvement in prenatal testing technology, an increasing number of children with cardiac tumors are being detected in the fetal period [9, 10]. Approximately 75% (18/24) of our cohort of 24 children were referred for medical examination, labor or other systemic diseases, and only a small proportion, approximately 25% (6/24), were referred for cardiovascular disease-related symptoms or signs. The clinical presentation of children with cardiac tumors varies due to tumor location, size, number, and the nature of the tumor. Its nonspecific manifestations often result in an incorrect or missed diagnosis and delayed time to optimal treatment, causing major complications or death. Therefore, prenatal testing is extremely important for timely diagnosis and treatment of primary cardiac tumors in children.

ECHO is the preferred test for cardiac tumors because of its ability to provide real-time data and its noninvasiveness, reproducibility, and high sensitivity [11]. It can detect the location, morphology, size, and mobility of cardiac tumors, as well as assess peripheral structures, such as congenital intracardiac malformations, outflow tract, and valves. ECHO plays an important role in the determination of the timing of surgical procedures and the elaboration of preoperative surgical protocols. However, ECHO also has shortcomings. Because of the differences in echocardiographic images of different types of tumors, there is a high concordance rate for the diagnosis of tumors in children with a high specificity but a low concordance rate for the diagnosis of tumors without a high specificity for ECHO. It needs to be evaluated in combination with other examinations. The determination of the nature of the tumor and identification of thrombus will also require further examination, such as MRI or CT [12]. Of course, pathologic examination is the gold standard for tumor diagnosis. In our cohort of cases, the concordance rate of ultrasound diagnosis of myxomas was approximately 90.91% (10/11), and the concordance rate of ultrasound diagnosis of tumors without high specificity was approximately 23.08% (3/13). There are reports in the literature that the concordance rate of ultrasound diagnosis of myxomas is 86.55% (103/119) but that the rate for others is 33.33% (3/9) [13].

The tumor can grow anywhere in the heart, one of the most important organs of the human body, and may involve the heart valves, coronary arteries, and conduction tracts, among other parts, causing obstruction, valvular stenosis or insufficiency, myocardial ischemia, and arrhythmias. Some tumor tissue sloughings cause systemic organ embolism and finally lead to heart failure, systemic organ dysfunction and even death. Therefore, regardless of the nature of the lesion, any lesion that occurs in the heart can have serious consequences, even the sudden death of the patient [14, 15]. In our series, there were 5 patients with lesions involving mitral or tricuspid valve tissues, 4 with outflow obstruction, and 3 with visceral embolization, and the patients underwent repair of diseased tissues at the same time during tumor resection surgery. The child with cerebral angio-embolism was treated with further rehabilitation after surgery with good results. Therefore, once cardiac neoplastic lesions are identified, they all require aggressive treatment, and some children need surgical resection. However, rhabdomyomas are unique among primary cardiac tumors. It has been reported in the literature that rhabdomyomas may be spontaneous regress [16]. In children with a high suspicion of rhabdomyoma on preoperative diagnosis, if the lesion temporarily has no significant hemodynamic effect, we suggest follow-up as appropriate. Otherwise, timely surgery is required.

The purpose of surgery for cardiac tumors is to restore normal hemodynamics, rather than completely removing the tumor. Removing the tumor can alleviate most of the arrhythmia [17], and there are literature reports that there is no significant difference in prognosis between complete or partial resection of tumors [18]. So we believe that as long as the reduced LV volume and affects hemodynamics, surgery is necessary for patients. For patients who are asymptomatic, routine follow-ups should be maintained. Complete resection should be performed for patients in which cardiac function is reserved without unstable hemodynamics or arrhythmia. Pacemaker is not required if arrhythmia is not observed. Otherwise, implantation of a pacemaker is necessary.

Primary cardiac tumors are mostly benign and less frequently malignant. Children without obvious symptoms can be closely followed up. Otherwise, prompt surgical treatment is needed, such as the presence of blood flow obstruction, embolization by the detachment of the tumor thrombus, arrhythmia, and valvular damage. The first attempt at the surgical resection of a cardiac tumor was made by Bahnson and Newman in 1953 [19], who blocked vena cava blood flow for a short time. Nearly 70 years have passed since Crafoord first successfully performed resection of a primary cardiac tumor in 1954 [20]. With the development of medical technology, surgical resection of primary cardiac tumors is quite mature today. We performed mostly a median thoracotomy to remove the tumor and could also complete the correction of intracardiac malformations in the same period. Some benign tumors may still recur, especially myxomas, which often have the highest recurrence rate due to incomplete excision [21]. Therefore, during resection of the cardiac tumor, it is necessary to remove the tumor as completely as possible, clean up the base, achieve a radical cure, and avoid recurrence [22]. However, the prognosis of malignant tumors is often associated with the pathological type. In this cohort, there were no recurrences after complete resection of the tumor in 21 children, and pathologic examination of the tumor in one child was suggestive of a borderline or malignant lesion. In this patient, who died on postoperative day 1, we considered that death was closely related to the patient's young age, poor preoperative base condition, intractable low cardiac output and rapid progression of the malignancy. We hope to be more experienced in dealing with similar cases in future. The base condition of the children should be adjusted as much as possible preoperatively, and the surgical protocol and postoperative emergency plans, such as routine backup ECMO, should be prepared in detail.

In summary, pediatric primary cardiac tumors are mostly benign lesions, and surgical treatment of pediatric primary cardiac tumors can obtain good results. However, it is necessary to fully evaluate the situation of cardiac tumors before the operation to make the best surgical plan and grasp the best surgical timing. For some asymptomatic children, it is not advisable to disrupt intact cardiac structures to resect the tumor completely. Hence, observation management during follow-up is suggested in these cases. However, prompt surgery is required to improve outcomes and avoid adverse outcomes for significantly symptomatic children.

## Data Availability

The authors confirm that the data supporting the findings of this study are available within the article.
